# Prognostic Role of Preoperative Vascular Cell Adhesion Molecule-1 Plasma Levels in Urothelial Carcinoma of the Bladder Treated With Radical Cystectomy

**DOI:** 10.1245/s10434-022-11575-4

**Published:** 2022-03-26

**Authors:** Keiichiro Mori, Victor M. Schuettfort, Satoshi Katayama, Ekaterina Laukhtina, Benjamin Pradere, Fahad Quhal, Reza Sari Motlagh, Hadi Mostafaei, Nico C. Grossmann, Pawel Rajwa, Jeremy YC. Teoh, Irene Resch, Harun Fajkovic, Marco Moschini, David D’andrea, Mohammad Abufaraj, Pierre I. Karakiewicz, Yair Lotan, Douglas Scherr, Shin Egawa, Eva Compérat, Shahrokh F. Shariat, European Association of Urology–Young Academic Urologists Urothelial Carcinoma Working Group (EAU-YAU)

**Affiliations:** 1 Department of Urology, Medical University of Vienna, Vienna, Austria; 2 Department of Urology, The Jikei University School of Medicine, Tokyo, Japan; 3 Department of Urology, University Medical Center Hamburg-Eppendorf, Hamburg, Germany; 4 Department of Urology, Dentistry and Pharmaceutical Sciences, Okayama University Graduate School of Medicine, Okayama, Japan; 5 Institute for Urology and Reproductive Health, Sechenov University, Moscow, Russia; 6 Department of Urology, CHRU Tours, Tours, France; 7 Université François Rabelais de Tours, PRES Centre Val de Loire, Tours, France; 8 Department of Urology, King Fahad Specialist Hospital, Dammam, Saudi Arabia; 9 Men’s Health and Reproductive Health Research Center, Shahid Beheshti University of Medical Sciences, Tehran, Iran; 10 Research Center for Evidence Based Medicine, Tabriz University of Medical Sciences, Tabriz, Iran; 11 Department of Urology, University Hospital Zurich, Zurich, Switzerland; 12 Department of Urology, Medical University of Silesia, Zabrze, Poland; 13 Department of Surgery, S.H. Ho Urology Centre, The Chinese University of Hong Kong, Hong Kong, China; 14 Klinik für Urologie, Luzerner Kantonsspital, Lucerne, Switzerland; 15 Hourani Center for Applied Scientific Research, Al-Ahliyya Amman University, Amman, Jordan; 16 Cancer Prognostics and Health Outcomes Unit, University of Montreal Health Centre, Montreal, Canada; 17 Department of Urology, University of Texas Southwestern, Dallas, TX USA; 18 Department of Urology, Weill Cornell Medical College, New York, NY USA; 19 Department of Pathology, Medical University of Vienna, Vienna, Austria; 20 Department of Urology, Second Faculty of Medicine, Charles University, Prague, Czech Republic; 21 Karl Landsteiner Institute of Urology and Andrology, Vienna, Austria

## Abstract

**Background:**

Angiogenesis-related marker vascular cell adhesion molecule-1 (VCAM-1) has been shown to be elevated in urothelial carcinoma of the bladder (UCB), but its predictive/prognostic role has not been determined. Thus, this study aimed to investigate the predictive/prognostic role of VCAM-1 for patients who have UCB treated with radical cystectomy (RC).

**Methods:**

The study enrolled 1036 patients with clinically non-metastatic advanced UCB who underwent RC, and plasma VCAM-1 was evaluated preoperatively. The correlation of plasma VCAM-1 with pathologic and survival outcomes was assessed using binominal logistic regression and multivariable Cox regression analyses. Discrimination was assessed using the area under the curve and concordance indices. The clinical net benefit was evaluated using decision curve analysis (DCA).

**Results:**

Preoperative VCAM-1 was significantly elevated in patients with adverse pathologic features. Higher VCAM-1 levels were independently associated with increased risk of lymph-node-metastasis (LNM), ≥pT3 disease, and non-organ-confined disease (NOCD (*p* < 0.001 for each). Preoperative plasma VCAM-1 was independently associated with recurrence-free survival (RFS), cancer-specific survival (CSS), and overall survival (OS) in pre- and postoperative multivariable models. Adding VCAM-1 to these predictive models improved their discriminatory ability to predict all outcomes by a significant margin. In the DCA, VCAM-1 addition to the reference models for prediction of LNM, NOCD, RFS, and CSS resulted in relevant improvement.

**Conclusions:**

Elevated plasma VCAM-1 was associated with biologically and clinically aggressive UCB disease features. After validation, preoperative VCAM-1 may serve as a biomarker to help identify patients likely to benefit from intensified/multimodal therapy. In addition, VCAM-1 improved the discriminatory power of predictive/prognostic models and can be used to refine personalized clinical decision-making.

**Supplementary Information:**

The online version contains supplementary material available at 10.1245/s10434-022-11575-4.

Radical cystectomy (RC) with lymph node dissection is the standard treatment for very-high-risk non-muscle-invasive and muscle-invasive urothelial carcinoma of the bladder (UCB).^1,2^ Despite definitive therapy with curative intent, the 5-year overall survival (OS) of patients remains below 60 %.^3,4^ Thus, various clinical and pathologic factors have been explored to improve the risk stratification of patients with UCB and to facilitate clinical decision-making and patient counseling.^5–7^

Unfortunately, the current outcome prediction models remain suboptimal, likely because of a failure to capture the full potential of host-tumor interactions.^8^ Additionally, clinical, radiologic, and pre-RC pathologic factors pose significant limitations for outcome prediction, thus limiting accurate personalized clinical decision-making.^5,9^ Identification of preoperative biomarkers capturing each tumor’s biologic and clinical potential is crucial to improvement of risk stratification for patients with UCB.^10^

Vascular cell adhesion molecule-1 (VCAM-1), which mediates cellular adhesion, is a 90-kDa transmembrane glycoprotein transiently expressed on the surface of different types of vascular endothelial and stromal cells in response to vascular endothelial growth factors (VEGFs) and cytokines.^11,12^ A close association exists between VCAM-1 and inflammation, immunologic disorders, tumor angiogenesis, and metastasis.^11,13^ Moreover, findings have shown that VCAM-1 is associated with oncologic outcomes in several cancers, with several studies reporting elevated serum VCAM-1 levels in patients with UCB.^14–16^ However, the predictive/prognostic value of blood VCAM-1 levels in patients who have UCB treated with RC remains uninvestigated.

We hypothesized that patients with clinically non-metastatic advanced UCB harboring occult metastases exhibit elevated plasma VCAM-1 levels, which are associated with features of aggressive disease and poor survival despite apparently effective disease control. We enrolled a large consecutive cohort of patients who had clinically non-metastatic advanced UCB treated with RC and pelvic lymphadenectomy to investigate the relationship between preoperative plasma VCAM-1 levels and established features of bladder cancer invasion, metastasis, and survival outcomes. Beyond multivariable modeling, we used predictive accuracy testing and decision curve analysis (DCA) to assess the value of preoperative VCAM-1 as a biomarker in real-world clinical scenarios.

## Material and Methods

### Patient Selection

All procedures were undertaken with the approval and oversight of the Institutional Review Board for the Protection of Human Subjects (1011011386 and 069826900). This retrospective study included a consecutive cohort of 1036 patients treated with RC for non-metastatic UCB between 2003 and 2015. No patient received neoadjuvant chemotherapy (NAC) or radiotherapy. Adjuvant chemotherapy (AC) was administered to 167 patients (16.1 %) at the clinicians' discretion based on tumor stage and overall health status.

### Measurement of Plasma VCAM-1 Levels

Preoperative plasma samples (subsequently used for VCAM-1 testing) were typically collected on the morning of surgery after an overnight fast. Their collection and measurement were performed as described previously.^12^ Briefly, blood was collected into 8-mL Vacutainer CPT tubes containing 0.1 mL of molar sodium citrate (Becton Dickinson Vacutainer Systems, Franklin Lakes, NJ, USA) and centrifuged at 1500 g for 20 min at room temperature. The top layer corresponding to plasma was decanted using sterile transfer pipettes. The plasma was immediately frozen and stored at −80 °C in polypropylene cryopreservation vials (Nalgene, Nalge Nunc, Rochester, NY, USA). Quantitative immunoassays were used to measure VCAM-1 levels (R&D systems, Minneapolis, MN, USA).

### Pathologic Evaluation and Patient Management

All surgical specimens were processed according to standard pathologic procedures as previously described.^17^ The 2002 American Joint Committee on Cancer tumor-node-metastasis (TNM) classification was used for pathologic staging, and the 1973 WHO/ISUP consensus classification was used for grading.

All the patients were followed up under the relevant institutional protocols and local guidelines. In general, the routine follow-up assessment included physical examination, radiologic imaging, and urine cytology every 3 months for 2 years. Between the second and fifth years, the follow-up assessment was performed semiannually, with follow-up evaluation subsequently performed annually in most cases. Recurrence was defined as any local recurrence (in the retroperitoneum or renal fossa) or distant metastasis. Recurrences in the bladder or contralateral upper urinary tract were considered as second primaries.

### Statistical Analysis

Categorical variables are reported as frequencies and proportions. Continuous coded variables are reported as medians and interquartile ranges (IQRs).

Given the non-normal distribution of the preoperative VCAM-1 levels, a log transformation was performed to reduce skewness and allow valid inference in the multivariable analysis. Patient characteristics and median preoperative plasma VCAM-1 levels were treated as categorical variables. Thus, group comparisons were performed using Mann-Whitney *U* or Kruskal-Wallis tests with subsequent significance testing as appropriate.

Binominal logistic regression analysis was performed to assess the association between preoperative VCAM-1 plasma levels and lymph node metastasis (LNM), ≥pT3 disease, or any non-organ-confined disease (NOCD, defined as ≥pT3 disease and/or LNM). In receiver operating characteristics (ROCs) curves, the area under the curve (AUC) was calculated to determine the predictive accuracy of the multiple logistic regression models. DeLong’s test was used to test the statistical significance between AUC differences.

Recurrence-free survival (RFS), cancer-specific survival (CSS), and OS were graphically visualized using the Kaplan-Meier method. The difference between groups was assessed using a log-rank test. Uni- and multivariable Cox regression models were used to investigate the associations of VCAM-1 with RFS, CSS, and OS. Clinical and pathologic tumor grades were excluded as variables for all predictive models because most patients had high-grade UCB. Separate Cox regression models that featured either preoperative clinical variables or postoperative histopathologic variables were developed. Their discriminatory ability and the additional information provided by plasma VCAM-1 levels were tested using Harrel’s concordance index (C-index).

The additional clinical net benefit of VCAM-1 also was evaluated using decision curve analysis (DCA) to investigate whether preoperative plasma VCAM-1 levels improved the accuracy of separate predictive and prognostic models and whether these models had a relevant net benefit in pre- or postoperative settings.^18^ Separate reference models that represented either the pre- or postoperative setting were created, to which VCAM-1 was added to assess the difference in predictive values. In addition, subgroup analyses were conducted among the patients with cT1, cT2, and pT2N0.

All *p* values were two-sided, and significance was defined as a *p* value lower than 0.05. Statistical analyses were performed using R version 3.6.3 (R Foundation for Statistical Computing, Vienna, Austria) and Stata/MP 14.2 statistical software (Stata Corp., College Station, TX, USA).

## Results

### Association Between Patient Characteristics and VCAM-1

Table 1 outlines the characteristics of the included patients. The median cohort age was 67 years (IQR, 60–73 years). The median plasma VCAM-1 levels were significantly higher for the patients with adverse pathologic features such as LNM and advanced pathologic tumor stage (*p* < 0.05).

**Table 1:** Patient demographics^a^

	Overall	Stratified by median log VCAM-1 levels		
Characteristic	(*n* = 1036) *n* (%)	Low VCAM-1 (*n* = 518) *n* (%)	High VCAM-1 (*n* = 518) *n* (%)	*p* Value
Median age: years (IQR)	67 (60–73)	66 (59–72)	67 (60–74)	0.10
Gender				0.02
Male	814 (79)	422 (81)	392 (76)	
Female	222 (21)	96 (19)	126 (24)	
Blood transfusion	268 (26)	122 (24)	146 (28)	0.09
Thrombocytosis	113 (11)	56 (11)	57 (11)	>0.9
Clinical tumor grade				>0.9
2	6 (0.6)	3 (0.6)	3 (0.6)	
3	1,022 (99)	512 (99)	510 (99)	
Unknown	8	3	5	
Clinical tumor stage				0.06
cTa	23 (2.2)	12 (2.3)	11 (2.1)	
cTis	105 (10)	42 (8.2)	63 (12)	
cT1	336 (33)	156 (30)	180 (35)	
cT2	498 (48)	268 (52)	230 (45)	
cT3	38 (3.7)	19 (3.7)	19 (3.7)	
cT4	29 (2.8)	18 (3.5)	11 (2.1)	
Unknown	7	3	4	
Pathologic tumor grade				0.8
1	62 (6.0)	29 (5.6)	33 (6.4)	
2	11 (1.1)	5 (1.0)	6 (1.2)	
3	963 (93)	484 (93)	479 (92)	
Pathologic tumor stage				<0.001
pT0	62 (6.0)	29 (5.6)	33 (6.4)	
pTa	22 (2.1)	9 (1.7)	13 (2.5)	
pTis	131 (13)	67 (13)	64 (12)	
pT1	162 (16)	104 (20)	58 (11)	
pT2	248 (24)	133 (26)	115 (22)	
pT3	281 (27)	122 (24)	159 (31)	
pT4	130 (13)	54 (10)	76 (15)	
Positive surgical margins	95 (9.2)	39 (7.5)	56 (11)	0.07
Lymphovascular invasion	295 (28)	138 (27)	157 (30)	0.2
Concomitant carcinoma *in situ*	572 (55)	309 (60)	263 (51)	0.004
Lymph node involvement	263 (25)	96 (19)	167 (32)	<0.001
Adjuvant chemotherapy	167 (16)	78 (15)	89 (17)	0.4

*VCAM-1* vascular cell adhesion molecule-1; *IQR* interquartile range

^a^Wilcoxon rank sum test; Pearson’s chi-squared test; Fisher’s exact test

### Association Between Advanced Pathologic Features and VCAM-1

**Fig. 1:**
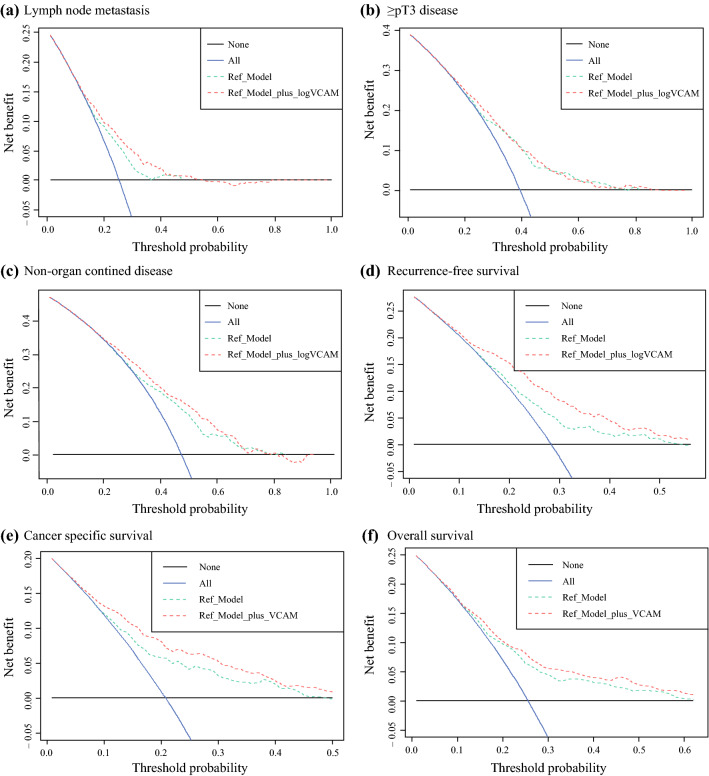
Decision curve analysis (DCA) for the benefit of preoperative vascular cell adhesion molecule 1 (VCAM-1) after inclusion in a preoperative model for advanced disease and survival prediction. The preoperative reference model consisted of age, sex, and clinical tumor stage, and the additional net benefit of preoperative VCAM-1 was investigated. **a** Lymph node metastasis. **b** ≥pT3 disease. **c** Non-organ-confined disease. **d** Recurrence-free survival. **e** Cancer-specific survival. **f** Overall survival. The x axis shows the threshold probabilities. The y axis measures the net benefit, calculated by adding the true-positives and subtracting the false-positives. The horizontal line representing the x axis assumes that no patients experience the event, whereas the grey line assumes that all patients will experience the event at a specific threshold probability. The dashed black line represents the net benefit of the reference Cox and logistic regression model. The dashed red line represents the net benefit of the same Cox and logistic regression models that also include the preoperative VCAM-1 plasma levels as a variable.

Multivariable logistic regression modeling showed significant association between elevated preoperative plasma VCAM-1 levels and an increased risk of LNM (odds ratio [OR], 2.25; 95 % confidence interval [CI], 1.72–2.97; *p* < 0.001), ≥pT3 disease (OR, 1.77; 95 % CI, 1.38–2.28; *p* < 0.001), and NOCD (OR, 1.77; 95 % CI, 1.38–2.28; *p* < 0.001) (Table 2). In the ROC curve analysis, addition of VCAM-1 to the reference model comprising age, sex, and clinical tumor stage improved its discriminatory ability to predict LNM (+5.8 %; *p* < 0.001), ≥pT3 disease (+2.0 %; *p* = 0.008), and NOCD (+4.4 %; *p* < 0.001). Addition of VCAM-1 to the reference model in DCA improved the net benefit for predicting LNM and NOCD (Fig. 1).

**Table 2:** Logistic regression modeling

	Lymph node involvement			pT3/4 disease			Any non-organ-confined disease		
Characteristic	OR	95 % CI	*p* Value	OR	95 % CI	*p* Value	OR	95 % CI	*p* Value
logVCAM-1	2.25	1.72–2.97	<0.001	1.77	1.38–2.28	<0.001	1.77	1.38–2.28	<0.001
Age	1.00	0.98–1.01	0.6	1.03	1.01–1.04	<0.001	1.03	1.01–1.04	<0.001
*Gender*									
Male	–	–		–	–		–	–	
Female	1.38	0.97–1.94	0.07	1.02	0.74–1.41	0.9	1.02	0.74–1.41	0.9
*Clinical tumor stage*									
cTa/cTis/cT1	–	–		–	–		–	–	
cT2	2.75	2.00–3.80	<0.001	2.91	2.20–3.87	<0.001	2.91	2.20–3.87	<0.001
cT3/cT4	3.74	2.11–6.56	<0.001	9.63	5.38–18.0	<0.001	9.63	5.38–18.0	<0.001
AUC with VCAM-1	0.687			0.695			0.720		
AUC without VCAM-1	0.629			0.675			0.676		

*OR* odds ratio, *CI* confidence interval, *VCAM-1* vascular cell adhesion molecule-1, *AUC* area under the curve

For the patients with cT1 and cT2, elevated preoperative plasma VCAM-1 levels also were significantly associated with an increased risk of LNM, ≥pT3 disease, and NOCD (Table S1). Adding VCAM-1 to the reference model improved the discriminatory ability to predict these outcomes.

### Association Between Survival Outcomes and VCAM-1 in the Preoperative Model

The median follow-up period for the alive patients was 37 months. The patients with higher median pretreatment VCAM-1 plasma levels had poorer RFS, CSS, and OS in the respective log-rank tests (all *p* < 0.001; Fig. S1).

In a multivariable Cox regression analysis that included available preoperative variables (i.e., age, sex, and clinical tumor stage), higher pretreatment VCAM-1 plasma levels were independently associated with poorer RFS (hazard ratio [HR], 2.90; 95 % CI, 2.46–3.41; *p* < 0.001), CSS (HR, 2.90; 95 % CI, 2.45–3.44; *p* < 0.001), and OS (HR, 1.77; 95 % CI, 1.52–2.05; *p* < 0.001) (Table 3). Adding the preoperative VCAM-1 plasma levels improved the C-indices of the same reference models for prognostication of RFS (+10.1 %), CSS (+8.7 %), and OS (+3.0 %). In the DCA, addition of VCAM-1 to the reference models for prognosis of RFS and CSS (Fig. 1) improved the net benefit of threshold probabilities from 10 % to 50 %.

**Table 3:** Multivariable Cox regression analysis that included available preoperative variables

	Recurrence-free survival			Cancer-specific survival			Overall survival		
Characteristic	HR	95 % CI	*p* Value	HR	95 % CI	*p* Value	HR	95 % CI	*p* Value
logVCAM-1	2.90	2.46–3.41	<0.001	2.90	2.45–3.44	<0.001	1.77	1.52–2.05	<0.001
Age	1.02	1.01–1.03	0.004	1.02	1.01–1.04	<0.001	1.05	1.04–1.06	<0.001
*Gender*									
Male	–	–		–	–		–	–	
Female	1.52	1.19–1.95	<0.001	1.64	1.27–2.12	<0.001	1.36	1.12–1.65	0.002
*Clinical tumor stage*									
cTa/cTis/cT1	–	–		–	–		–	–	
cT2	1.96	1.55–2.47	<0.001	2.09	1.63–2.69	<0.001	1.72	1.44–2.06	<0.001
cT3/cT4	2.17	1.44–3.28	<0.001	2.38	1.56–3.64	<0.001	1.98	1.44–2.73	<0.001
C-index with VCAM-1	0.703			0.715			0.664		
C-index without VCAM-1	0.602			0.628			0.634		

*HR* hazard ratio, *CI* confidence interval, *VCAM-1* vascular cell adhesion molecule-1

For the patients with cT1 and cT2, higher pretreatment plasma VCAM-1 levels also were independently associated with poorer RFS, CSS, and OS (Table S2). Adding the preoperative plasma VCAM-1 levels improved the C-indices of the same reference models for the prediction of all these outcomes.

### Association Between Survival Outcomes and VCAM-1 in the Postoperative Model

**Fig. 2:**
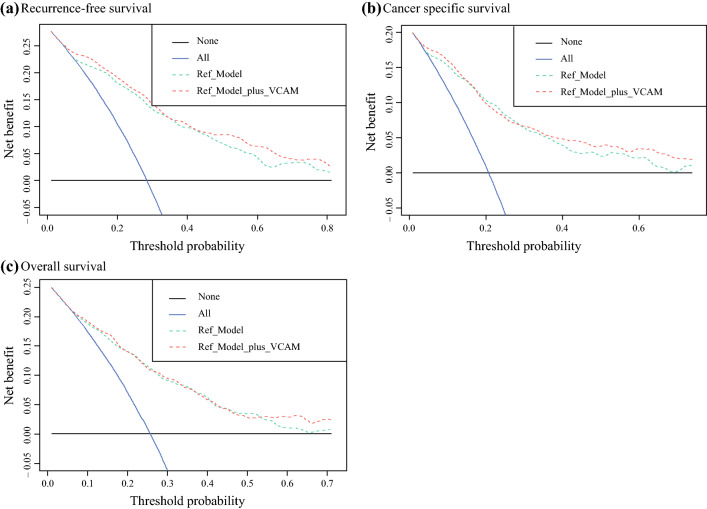
Decision curve analysis (DCA) for the preoperative vascular cell adhesion molecule 1 (VCAM-1) benefit after inclusion in a postoperative model for survival prediction. The postoperative reference model consisted of established postoperative variables for prediction of recurrence-free survival, cancer-specific survival, and overall survival, and the additional net benefit of preoperative VCAM-1 was investigated. **a** Recurrence-free survival. **b** Cancer-specific survival. **c** Overall survival. The x axis shows the threshold probabilities. The y axis measures the net benefit, calculated by adding the true-positives and subtracting the false-positives. The horizontal line representing the x axis assumes that no patients experience the event, whereas the grey line assumes that all patients will experience the event at a specific threshold probability. The dashed black line represents the net benefit of the reference Cox regression model. The dashed red line represents the net benefit of the same Cox regression models that also include the preoperative VCAM-1 plasma levels as a variable.

In a multivariable Cox regression model including established postoperative variables, higher pretreatment VCAM-1 plasma levels remained independently associated with poorer RFS (HR, 2.53; 95 % CI, 2.12–3.02; *p* < 0.001), CSS (HR, 2.49; 95 % CI, 2.06–3.00; *p* < 0.001), and OS (HR, 1.48; 95 % CI, 1.26–1.73; *p* < 0.001) (Table 4). Adding the VCAM-1 to the reference models (Table 4) improved the C-indices for prognostication of RFS (+3.2 %), CSS (+2.5 %), and OS (+<1 %). In the DCA, including the preoperative plasma VCAM-1 levels resulted in a net benefit of threshold probabilities from 50 % to 70 % for predicting RFS, from 40 % to 70 % for predicting CSS, and from 60 % to 70 % for predicting OS relative to the reference model (Fig. 2).

**Table 4:** Multivariable Cox regression model that included established postoperative variables

	Recurrence-free survival			Cancer-specific survival			Overall survival		
Characteristic	HR	95 % CI	*p* Value	HR	95 % CI	*p* Value	HR	95 % CI	*p* Value
logVCAM-1	2.53	2.12–3.02	<0.001	2.49	2.06–3.00	<0.001	1.48	1.26–1.73	<0.001
Age	1.01	1.00–1.02	0.13	1.02	1.00–1.03	0.009	1.04	1.03–1.05	<0.001
*Gender*									
Male	–	–		–	–		–	–	
Female	1.64	1.28–2.10	<0.001	1.70	1.31–2.19	<0.001	1.40	1.15–1.70	<0.001
*Pathologic tumor stage*									
pT0/pTa/pTis/pT1	–	–		–	–		–	–	
pT2	1.42	0.98–2.07	0.06	1.39	0.93–2.07	0.11	1.45	1.14–1.85	0.003
pT3/pT4	3.14	2.23–4.43	<0.001	2.99	2.08–4.31	<0.001	2.50	1.96–3.19	<0.001
Positive surgical margins	1.47	1.08–2.00	0.02	1.60	1.16–2.20	0.004	1.14	0.86–1.52	0.3
Lymphovascular invasion	1.43	1.12–1.84	0.005	1.59	1.22–2.06	<0.001	1.23	1.01–1.50	0.041
Concomitant carcinoma *in situ*	1.20	0.95–1.50	0.12	1.04	0.82–1.32	0.7	1.06	0.89–1.26	0.5
Lymph node involvement	2.25	1.75–2.89	<0.001	2.29	1.76–2.97	<0.001	1.96	1.59–2.41	<0.001
Adjuvant chemotherapy	0.96	0.73–1.26	0.8	1.02	0.76–1.35	>0.9	0.87	0.69–1.10	0.3
C-index with VCAM-1	0.785			0.801			0.740		
C-index without VCAM-1	0.753			0.776			0.735		

*HR* hazard ratio, *CI* confidence interval, *VCAM-1* vascular cell adhesion molecule-1

For the patients with cT1 and cT2, higher pretreatment plasma VCAM-1 levels were independently associated with poorer RFS, CSS, and OS (Table S3). For the patients with pT2N0, higher pretreatment VCAM-1 plasma levels were independently associated with poorer RFS and CSS. Adding the VCAM-1 to the reference models improved the C-indices for prognostication of these outcomes.

## Discussion

Although biomarkers help improve risk stratification by estimating the probability of treatment failure, clinically useful biomarkers for UCB are lacking.^19,20^ Therefore, we assessed the prognostic significance of preoperative plasma VCAM-1 levels in a large cohort of patients who had UCB treated with RC. We demonstrated that elevated preoperative VCAM-1 levels were associated with worse oncologic outcomes and predicted biologically and clinically aggressive disease. These findings suggest that blood levels of VCAM-1 could help facilitate clinical decision-making regarding perioperative chemotherapy and the extent of lymphadenectomy together with patient counseling.

The mechanism underlying the association of VCAM-1 with survival may be its relationship with tumor angiogenesis and metastasis. Serum VCAM-1 levels were observed to correlate with micro vessel density in breast cancer, suggesting that serum VCAM-1 is a surrogate angiogenesis marker.^21^ Furthermore, VEGF has been shown to upregulate VCAM-1 expression in endothelial cells.^22,23^ Additionally, there is an interaction between VCAM-1 and α4β1 integrin during angiogenesis. Findings have shown that VCAM-1 and α4β1 integrin are individually expressed on vascular smooth muscle cells and endothelial cells in the developing vessels of breast cancer.

Administration of an anti-murine VCAM-1 antibody reduced micro vessel formation in Matrigel plug mouse models.^24^ Furthermore, *in vitro* exposure to anti-VCAM-1 antibody blocked interleukin (IL)-4 and IL-13-induced tube formation, and angiogenesis induced *in vivo* by IL-4 and IL-13 was inhibited by an anti-α4 integrin antibody.^25^

Expression of VCAM-1 also is associated with tumor metastasis.^11,13^ A comparative gene profile analysis of parental MDA-MB-231 breast cancer cells and *in vivo* isolates exhibiting lung metastatic activity showed VCAM-1 expression to be upregulated in metastatic breast cancer cells in the lungs.^26^ Furthermore, mesothelial VCAM-1 expression was negatively associated with RFS and OS in epithelial ovarian cancer.^14^ Studies also have shown that expression of VCAM-1 is upregulated in lung and colorectal cancers.^27, 28^ Taken together, cumulative data suggested that increased VCAM-1 enhances tumor angiogenesis, adhesion, and metastasis, leading to advanced disease and increased cancer-related mortality.

To assess whether VCAM-1 can improve the prognostic outcome of established predictors requires more than conventional multivariable analyses.^19,20^ Analyzing the potential of a novel biomarker to enhance the predictive/prognostic reference model enables us to determine whether the biomarker itself holds unique predictive/prognostic information.^19,20^ Therefore, we examined whether preoperative VCAM-1 plasma levels improved the accuracy of separate predictive/prognostic models and whether these models had a relevant net benefit in pre- or postoperative settings using DCA. Indeed, we found that addition of VCAM-1 to standard models improved the discriminatory power for predicting LNM, ≥pT3 disease, and NOCD by a statistically and prognostically significant margin. Furthermore, VCAM-1 also considerably improved prognosis of early RFS and CSS. In the DCA, VCAM-1 addition improved the clinical net benefit of several reference models in a wide range of threshold probabilities, suggesting its clinical potential for guiding preoperative risk stratification through improved early outcome prediction. In essence, plasma VCAM-1 seems to improve patient selection for intensified and/or multimodal perioperative systemic therapy with the goal of prognosis.

In addition, VCAM-1 demonstrated the potential to improve outcome prediction if added to a model consisting of established histopathologic variables. In this postoperative setting, our prognostic model exhibited a high discriminatory power (C-Index of 0.79 for RFS prediction and 0.80 for CSS prediction), which could allow for tailored therapy, thereby improving patient care. In the DCA, VCAM-1 addition slightly improved the net benefit of this model for prediction of both RFS and CSS.

Guidelines recommend the use of NAC for cT2-4aN0M0 and the use of AC for pT3/4 and/or pN1 UCB.^1^ Of clinical interest is whether NAC should be offered for only cT2-4aN0M0 and AC for only pT3/4 and/or pN1, or whether NAC should be offered to all patients with cT2. Therefore, subgroup analyses were performed among cT1, cT2, and pT2N0 patients to assess the role of perioperative chemotherapy. Subsequently, in all these cohorts, adding VCAM-1 to the reference models improved the discriminatory ability to predict LNM, ≥pT3 disease, NOCD, RFS, and CSS by a prognostically significant margin. These results suggested that cT1 patients with elevated VCAM-1 also may benefit from intensified therapy such as NAC because they are more likely to be upstaged at the time of RC. Similarly, pT2N0 patients with elevated VCAM-1 also may benefit from AC because they are more likely to harbor micrometastatic disease. Moreover, in cT2, preoperative VCAM-1 also might serve as a biomarker to identify patients who could avoid NAC because the risk for RFS/CSS is very low. However, caution should be exercised in interpreting these results given that these subgroup analyses had less statistical power.

Although this was the largest study to investigate the predictive/prognostic value of VCAM-1, it had limitations. First, its retrospective and multicenter design may have resulted in laboratory, pathologic, and surgical workup variations, thus confounding the results. Second, the VCAM-1 value was assessed at a single time point preoperatively and not evaluated for its variability over time. Third, because of the recruitment time for this study, no information was available on the predictive value of VCAM-1 with respect to immunotherapies or NAC. Finally, although next-generation sequencing and immunohistology have shown several other candidate biomarkers, the results often are limited in reproducibility because of cost, intratumor heterogeneity, absence of a standardized approach, and overall complexity,^29,30^ hindering their implementation into clinical practice. Therefore, given the ease of procurement, low cost, high sample homogeneity, and the potential to improve early outcome prediction and prognosis, VCAM-1 warrants further evaluation as a candidate biomarker for integration into prospective clinical trials.^19^

## Conclusion

To our knowledge, this is the first study to investigate VCAM-1 plasma levels in patients who have UCB treated with RC. Elevated plasma VCAM-1 levels were associated with features of biologically and clinically aggressive disease in patients with UCB. The use of VCAM-1 improved the discriminatory power of predictive/prognostic models by a significant margin. Therefore, VCAM-1 might be a valuable biomarker to guide physicians on the need for perioperative chemotherapy and the extent of lymphadenectomy. Thus, well-designed prospective studies with adequate follow-up evaluation are warranted to validate the use of VCAM-1 as a biomarker to enhance current tools used for risk stratification of patients with UCB and to assess its value in this age of immunotherapy.

## Supplementary Information

Below is the link to the electronic supplementary material.

Supplementary file1 (DOCX 24 kb)

Supplementary file2 (DOCX 25 kb)

Supplementary file3 (DOCX 35 kb)

Supplementary file4 (PDF 118 kb)

Supplementary file5 (DOCX 40 kb)
