# Supplementary material for: Prognostic Role of Preoperative Vascular Cell Adhesion Molecule-1 Plasma Levels in Urothelial Carcinoma of the Bladder Treated With Radical Cystectomy
Source: Ann Surg Oncol. 2022 Mar 26;29(8):5307–16. doi: 10.1245/s10434-022-11575-4 (PMC9246812; doi:10.1245/s10434-022-11575-4)
Supplement: Supplementary file 1 — Supplementary file1 (DOCX 24 kb) [file 10434_2022_11575_MOESM1_ESM.docx]

Supplementary Table 1 Logistic regression modeling

1. cT1

|  | Lymph node involvement | | | pT3/4 disease | | | Any non-organ confined disease | | |
| --- | --- | --- | --- | --- | --- | --- | --- | --- | --- |
| Characteristic | OR | 95% CI | p-value | OR | 95% CI | p-value | OR | 95% CI | p-value |
| logVCAM-1 | 2.29 | 1.31, 4.00 | 0.003 | 2.51 | 1.54, 4.08 | <0.001 | 2.92 | 1.81, 4.73 | <0.001 |
| Age | 0.98 | 0.96, 1.01 | 0.29 | 1.01 | 0.99, 1.04 | 0.28 | 1.00 | 0.98, 1.03 | 0.84 |
| Gender |  |  |  |  |  |  |  |  |  |
| male | — | — |  | — | — |  | — | — |  |
| female | 1.33 | 0.67, 2.63 | 0.41 | 0.85 | 0.47, 1.54 | 0.59 | 1.06 | 0.61, 1.86 | 0.83 |
| AUC with VCAM-1 | 0.644 | | | 0.640 | | | 0.660 | | |
| AUC without VCAM-1 | 0.550 | | | 0.555 | | | 0.515 | | |
| OR = Odds Ratio, CI = Confidence Interval, AUC = area under the curve | | | | | | | | | |

1. cT2

|  | Lymph node involvement | | | pT3/4 disease | | | Any non-organ confined disease | | |
| --- | --- | --- | --- | --- | --- | --- | --- | --- | --- |
| Characteristic | OR | 95% CI | p-value | OR | 95% CI | p-value | OR | 95% CI | p-value |
| logVCAM-1 | 2.16 | 1.52, 3.08 | <0.001 | 1.38 | 1.00, 1.91 | 0.047 | 2.12 | 1.49, 3.00 | <0.001 |
| Age | 1.00 | 0.98, 1.02 | 0.98 | 1.03 | 1.01, 1.05 | 0.003 | 1.03 | 1.01, 1.04 | 0.009 |
| Gender |  |  |  |  |  |  |  |  |  |
| male | — | — |  | — | — |  | — | — |  |
| female | 1.19 | 0.75, 1.89 | 0.46 | 1.06 | 0.69, 1.64 | 0.79 | 1.13 | 0.72, 1.78 | 0.59 |
| AUC with VCAM-1 | 0.638 | | | 0.589 | | | 0.644 | | |
| AUC without VCAM-1 | 0.508 | | | 0.571 | | | 0.570 | | |
| OR = Odds Ratio, CI = Confidence Interval, AUC = area under the curve | | | | | | | | | |
