# Supplementary material for: Prognostic Role of Preoperative Vascular Cell Adhesion Molecule-1 Plasma Levels in Urothelial Carcinoma of the Bladder Treated With Radical Cystectomy
Source: Ann Surg Oncol. 2022 Mar 26;29(8):5307–16. doi: 10.1245/s10434-022-11575-4 (PMC9246812; doi:10.1245/s10434-022-11575-4)
Supplement: Supplementary file 2 — Supplementary file2 (DOCX 25 kb) [file 10434_2022_11575_MOESM2_ESM.docx]

Supplementary Table 2 Multivariable Cox regression analysis that included available preoperative variables

1. cT1

|  | Recurrence-free Survival | | | Cancer-specific survival | | | Overall survival | | |
| --- | --- | --- | --- | --- | --- | --- | --- | --- | --- |
| Characteristic | HR | 95% CI | p-value | HR | 95% CI | p-value | HR | 95% CI | p-value |
| logVCAM-1 | 2.89 | 2.03, 4.02 | <0.001 | 2.93 | 2.02, 4.15 | <0.001 | 1.76 | 1.30, 2.36 | <0.001 |
| Age | 1.03 | 1.00, 1.05 | 0.02 | 1.03 | 1.01, 1.06 | 0.01 | 1.05 | 1.03, 1.07 | <0.001 |
| Gender |  |  |  |  |  |  |  |  |  |
| Male | — | — |  | — | — |  | — | — |  |
| Female | 1.36 | 0.81, 2.20 | 0.24 | 1.59 | 0.93, 2.62 | 0.09 | 1.14 | 0.75, 1.66 | 0.53 |
| C-index with VCAM-1 | 0.688 | | | 0.699 | | | 0.645 | | |
| C-index without VCAM-1 | 0.571 | | | 0.595 | | | 0.608 | | |
| HR = Hazard Ratio, CI = Confidence Interval | | | | | | | | | |

1. cT2

|  | Recurrence-free Survival | | | Cancer-specific survival | | | Overall survival | | |
| --- | --- | --- | --- | --- | --- | --- | --- | --- | --- |
| Characteristic | HR | 95% CI | p-value | HR | 95% CI | p-value | HR | 95% CI | p-value |
| logVCAM-1 | 2.80 | 2.28, 3.43 | <0.001 | 2.76 | 2.23, 3.41 | <0.001 | 1.69 | 1.39, 2.04 | <0.001 |
| Age | 1.02 | 1.00, 1.03 | 0.03 | 1.02 | 1.00, 1.03 | 0.041 | 1.04 | 1.03, 1.06 | <0.001 |
| Gender |  |  |  |  |  |  |  |  |  |
| Male | — | — |  | — | — |  | — | — |  |
| Female | 1.46 | 1.03, 2.03 | 0.09 | 1.60 | 1.12, 2.24 | 0.008 | 1.40 | 1.07, 1.82 | 0.01 |
| C-index with VCAM-1 | 0.706 | | | 0.703 | | | 0.647 | | |
| C-index without VCAM-1 | 0.562 | | | 0.577 | | | 0.612 | | |
| HR = Hazard Ratio, CI = Confidence Interval | | | | | | | | | |
