# Supplementary material for: Prognostic Role of Preoperative Vascular Cell Adhesion Molecule-1 Plasma Levels in Urothelial Carcinoma of the Bladder Treated With Radical Cystectomy
Source: Ann Surg Oncol. 2022 Mar 26;29(8):5307–16. doi: 10.1245/s10434-022-11575-4 (PMC9246812; doi:10.1245/s10434-022-11575-4)
Supplement: Supplementary file 3 — Supplementary file3 (DOCX 35 kb) [file 10434_2022_11575_MOESM3_ESM.docx]

Supplementary Table 3

1. cT1

|  | Recurrence-free Survival | | | Cancer-specific survival | | | Overall survival | | |
| --- | --- | --- | --- | --- | --- | --- | --- | --- | --- |
| Characteristic | HR | 95% CI | p-value | HR | 95% CI | p-value | HR | 95% CI | p-value |
| logVCAM-1 | 2.64 | 1.83, 3.79 | <0.001 | 2.81 | 1.91, 4.13 | <0.001 | 1.82 | 1.34, 2.44 | <0.001 |
| Age | 1.03 | 1.00, 1.05 | 0.04 | 1.04 | 1.01, 1.07 | 0.01 | 1.05 | 1.03, 1.07 | <0.001 |
| Gender |  |  |  |  |  |  |  |  |  |
| male | — | — |  | — | — |  | — | — |  |
| female | 1.49 | 0.88, 2.43 | 0.13 | 1.71 | 0.98, 2.87 | 0.048 | 1.26 | 0.83, 1.87 | 0.26 |
| Pathological tumor stage |  |  |  |  |  |  |  |  |  |
| pT0/pTa/pTis/pT1 | — | — |  | — | — |  | — | — |  |
| pT2 | 2.06 | 1.06, 3.97 | 0.03 | 1.79 | 0.88, 3.61 | 0.11 | 1.91 | 1.20, 2.99 | 0.006 |
| pT3/pT4 | 3.53 | 1.77, 7.05 | <0.001 | 2.53 | 1.18, 5.42 | 0.02 | 2.73 | 1.56, 4.74 | <0.001 |
| Positive surgical margins | 0.76 | 0.35, 1.47 | 0.44 | 1.01 | 0.46, 2.01 | 0.98 | 0.78 | 0.42, 1.36 | 0.40 |
| Lymphovascular invasion | 1.75 | 1.04, 2.90 | 0.03 | 2.32 | 1.33, 3.98 | 0.003 | 1.94 | 1.25, 2.95 | 0.002 |
| Concomitant Carcinoma in situ | 1.78 | 1.08, 2.95 | 0.02 | 1.23 | 0.71, 2.13 | 0.46 | 1.54 | 1.03, 2.31 | 0.036 |
| Lymph node involvement | 2.46 | 1.45, 4.09 | <0.001 | 3.31 | 1.88, 5.79 | <0.001 | 2.43 | 1.48, 3.90 | <0.001 |
| Adjuvant chemotherapy | 1.58 | 0.86, 2.74 | 0.12 | 1.56 | 0.83, 2.80 | 0.15 | 1.19 | 0.70, 1.93 | 0.51 |
| C-index with VCAM-1 | 0.788 | | | 0.812 | | | 0.748 | | |
| C-index without VCAM-1 | 0.756 | | | 0.783 | | | 0.738 | | |
| HR = Hazard Ratio, CI = Confidence Interval | | | | | | | | | |

1. cT2

|  | Recurrence-free Survival | | | Cancer-specific survival | | | Overall survival | | |
| --- | --- | --- | --- | --- | --- | --- | --- | --- | --- |
| Characteristic | HR | 95% CI | p-value | HR | 95% CI | p-value | HR | 95% CI | p-value |
| logVCAM-1 | 2.75 | 2.18, 3.48 | <0.001 | 2.73 | 2.13, 3.51 | <0.001 | 1.49 | 1.21, 1.83 | <0.001 |
| Age | 1.00 | 0.99, 1.02 | 0.94 | 1.01 | 0.99, 1.02 | 0.38 | 1.04 | 1.02, 1.05 | <0.001 |
| Gender |  |  |  |  |  |  |  |  |  |
| male | — | — |  | — | — |  | — | — |  |
| female | 1.61 | 1.13, 2.25 | 0.007 | 1.66 | 1.16, 2.34 | 0.005 | 1.47 | 1.12, 1.90 | 0.005 |
| Pathological tumor stage |  |  |  |  |  |  |  |  |  |
| pT0/pTa/pTis/pT1 | — | — |  | — | — |  | — | — |  |
| pT2 | 1.16 | 0.65, 2.14 | 0.61 | 1.13 | 0.61, 2.17 | 0.69 | 1.45 | 1.02, 2.08 | 0.039 |
| pT3/pT4 | 3.35 | 1.98, 5.93 | <0.001 | 3.22 | 1.85, 5.92 | <0.001 | 2.62 | 1.84, 3.77 | <0.001 |
| Positive surgical margins | 1.85 | 1.14, 2.90 | 0.009 | 1.86 | 1.12, 2.99 | 0.01 | 1.29 | 0.80, 1.98 | 0.28 |
| Lymphovascular invasion | 1.22 | 0.87, 1.71 | 0.25 | 1.32 | 0.93, 1.88 | 0.12 | 1.02 | 0.78, 1.33 | 0.88 |
| Concomitant Carcinoma in situ | 1.05 | 0.78, 1.43 | 0.73 | 1.02 | 0.75, 1.40 | 0.90 | 1.08 | 0.85, 1.36 | 0.54 |
| Lymph node involvement | 2.01 | 1.41, 2.87 | <0.001 | 1.93 | 1.33, 2.79 | <0.001 | 1.75 | 1.31, 2.35 | <0.001 |
| Adjuvant chemotherapy | 0.84 | 0.58, 1.21 | 0.36 | 0.94 | 0.65, 1.36 | 0.74 | 0.85 | 0.63, 1.16 | 0.30 |
| C-index with VCAM-1 | 0.775 | | | 0.780 | | | 0.719 | | |
| C-index without VCAM-1 | 0.732 | | | 0.749 | | | 0.712 | | |
| HR = Hazard Ratio, CI = Confidence Interval | | | | | | | | | |

1. pT2N0

|  | Recurrence-free Survival | | | Cancer-specific survival | | | Overall survival | | |
| --- | --- | --- | --- | --- | --- | --- | --- | --- | --- |
| Characteristic | HR | 95% CI | p-value | HR | 95% CI | p-value | HR | 95% CI | p-value |
| logVCAM-1 | 2.81 | 1.80, 4.39 | <0.001 | 2.55 | 1.56, 4.21 | <0.001 | 1.18 | 0.82, 1.69 | 0.37 |
| Age | 1.04 | 1.00, 1.09 | 0.042 | 1.05 | 1.01, 1.11 | 0.02 | 1.08 | 1.06, 1.11 | <0.001 |
| Gender |  |  |  |  |  |  |  |  |  |
| male | — | — |  | — | — |  | — | — |  |
| female | 1.36 | 0.57, 2.96 | 0.47 | 1.74 | 0.72, 3.91 | 0.21 | 1.58 | 0.93, 2.58 | 0.08 |
| Positive surgical margins | 0.41 | 0.02, 2.02 | 0.33 | 0.46 | 0.03, 2.29 | 0.40 | 0.45 | 0.11, 1.23 | 0.18 |
| Lymphovascular invasion | 2.34 | 1.01, 5.25 | 0.048 | 3.24 | 1.29, 7.91 | 0.01 | 2.23 | 1.35, 3.60 | 0.001 |
| Concomitant Carcinoma in situ | 0.97 | 0.47, 2.09 | 0.94 | 0.74 | 0.33, 1.68 | 0.46 | 0.99 | 0.64, 1.57 | 0.98 |
| Adjuvant chemotherapy | 0.80 | 0.13, 2.76 | 0.76 | 0.93 | 0.15, 3.28 | >0.9 | 0.50 | 0.17, 1.19 | 0.16 |
| C-index with VCAM-1 | 0.792 | | | 0.786 | | | 0.693 | | |
| C-index without VCAM-1 | 0.690 | | | 0.718 | | | 0.689 | | |
| HR = Hazard Ratio, CI = Confidence Interval | | | | | | | | | |
