# Supplementary material for: Prognostic Role of Preoperative Vascular Cell Adhesion Molecule-1 Plasma Levels in Urothelial Carcinoma of the Bladder Treated With Radical Cystectomy
Source: Ann Surg Oncol. 2022 Mar 26;29(8):5307–16. doi: 10.1245/s10434-022-11575-4 (PMC9246812; doi:10.1245/s10434-022-11575-4)
Supplement: Supplementary file 4 — Supplementary file4 (PDF 118 kb) [file 10434_2022_11575_MOESM4_ESM.pdf]

Supplementary Figure 1

Kaplan Meier estimates of oncological outcomes stratified by VCAM-1 in 1036 patients with urothelial carcinoma of the bladder (UCB) treated with radical cystectomy (RC)

(A) Recurrence-free survival

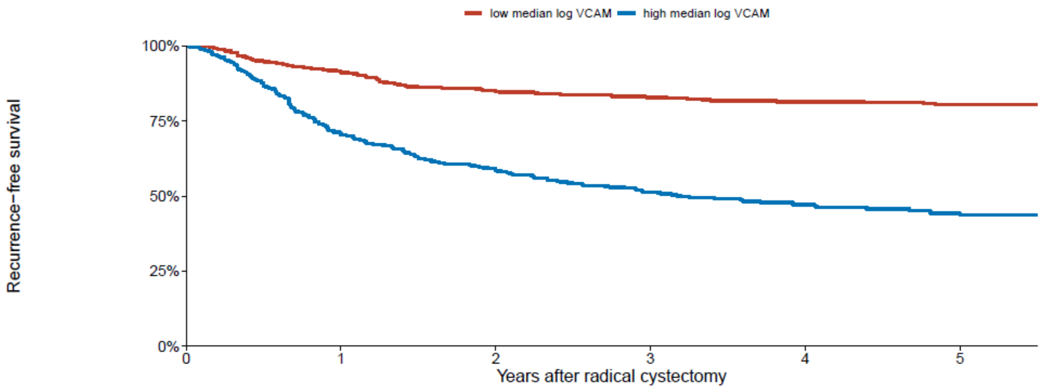

Number at risk

low median log VCAM	518	401	319	294	266	244
high median log VCAM	518	324	236	164	114	84

Cumulative number of events

low median log VCAM	0	42	68	75	80	83
high median log VCAM	0	144	194	220	232	239

Cumulative number of censoring

low median log VCAM	0	79	133	149	174	192
high median log VCAM	0	57	93	135	177	197

(B) Cancer-specific survival

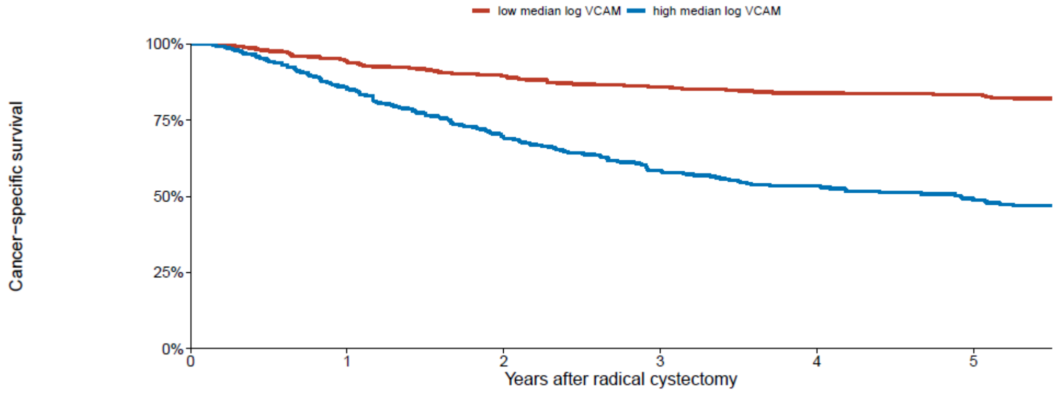

Number at risk

low median log VCAM	518	413	341	307	276	254
high median log VCAM	518	392	282	193	135	101

Cumulative number of events

low median log VCAM	0	27	46	59	66	68
high median log VCAM	0	71	139	178	193	203

Cumulative number of censoring

low median log VCAM	4	83	132	152	178	197
high median log VCAM	2	63	103	148	195	215

(C) Overall survival

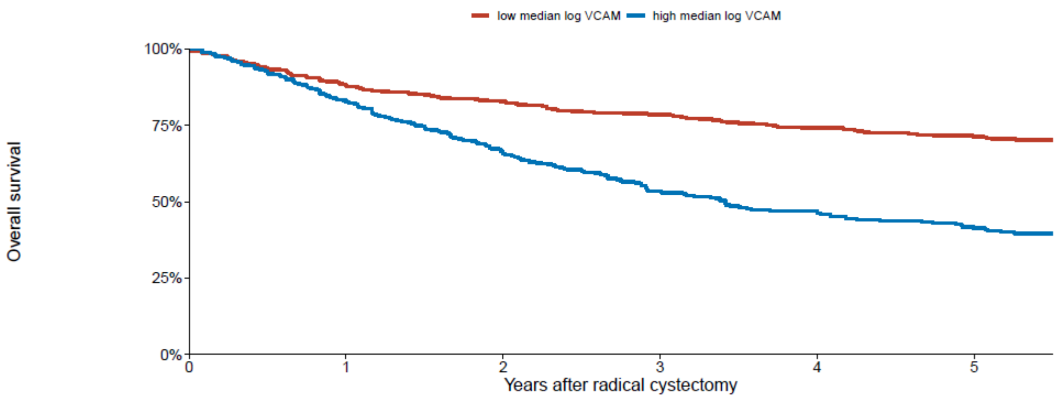

Number at risk

	0	1	2	3	4	5
low median log VCAM	518	413	341	307	276	254
high median log VCAM	518	392	282	193	135	101

Cumulative number of events

	0	1	2	3	4	5
low median log VCAM	3	59	82	99	116	126
high median log VCAM	0	86	160	208	231	244

Cumulative number of censoring

	0	1	2	3	4	5
low median log VCAM	1	51	96	112	128	139
high median log VCAM	2	48	82	118	157	174
